# SelANet: decision-assisting selective sleep apnea detection based on confidence score

**DOI:** 10.1186/s12911-023-02292-3

**Published:** 2023-09-21

**Authors:** Beomjun Bark, Borum Nam, In Young Kim

**Affiliations:** 1 Department of Biomedical Engineering, Hanyang University, 222, Wangsimni-Ro, Seongdong-Gu, 04763 Seoul, Republic of Korea; 2 Department of Electronic Engineering, Hanyang University, Seoul, Republic of Korea

**Keywords:** Artificial intelligence, Sleep apnea syndrome, Selective prediction, Decision assisting, Wearable devices

## Abstract

**Background:**

One of the most common sleep disorders is sleep apnea syndrome. To diagnose sleep apnea syndrome, polysomnography is typically used, but it has limitations in terms of labor, cost, and time. Therefore, studies have been conducted to develop automated detection algorithms using limited biological signals that can be more easily diagnosed. However, the lack of information from limited signals can result in uncertainty from artificial intelligence judgments. Therefore, we performed selective prediction by using estimated respiratory signals from electrocardiogram and oxygen saturation signals based on confidence scores to classify only those sleep apnea occurrence samples with high confidence. In addition, for samples with high uncertainty, this algorithm rejected them, providing a second opinion to the clinician.

**Method:**

Our developed model utilized polysomnography data from 994 subjects obtained from Massachusetts General Hospital. We performed feature extraction from the latent vector using the autoencoder. Then, one dimensional convolutional neural network—long short-term memory (1D CNN-LSTM) was designed and trained to measure confidence scores for input, with an additional selection function. We set a confidence score threshold called the target coverage and performed optimization only on samples with confidence scores higher than the target coverage. As a result, we demonstrated that the empirical coverage trained in the model converged to the target coverage.

**Result:**

To confirm whether the model has been optimized according to the objectives, the coverage violation was used to measure the difference between the target coverage and the empirical coverage. As a result, the value of coverage violation was found to be an average of 0.067. Based on the model, we evaluated the classification performance of sleep apnea and confirmed that it achieved 90.26% accuracy, 91.29% sensitivity, and 89.21% specificity. This represents an improvement of approximately 7.03% in all metrics compared to the performance achieved without using a selective prediction.

**Conclusion:**

This algorithm based on selective prediction utilizes confidence measurement method to minimize the problem caused by limited biological information. Based on this approach, this algorithm is applicable to wearable devices despite low signal quality and can be used as a simple detection method that determine the need for polysomnography or complement it.

## Background

Sleep apnea is a type of sleep breathing disorder in which abnormal breathing patterns occur during sleep [1]. The prevalence of sleep apnea syndrome is up to 15–30% for men and 10–15% for women in North America, indicating that it affects many people [2]. Not only does sleep apnea cause poor sleep quality, but it can also lead to high blood pressure, headaches, depression, and other problems if the symptoms persist [3]. It can also cause cardiovascular problems and even sudden death [4]. The standard method for diagnosing sleep apnea syndrome is polysomnography [5]. Polysomnography is a test that measures a variety of biological signals during a night’s sleep in a sleep center. Sleep apnea diagnosis relies on a variety of bio-measurements, such as EEG, nasal pressure cannula, and pulse oximetry, which are measured during polysomnography [6, 7]. Also, using these bio-signals, polysomnography is used to estimate the apnea hypopnea index (AHI) to quantify sleep apnea syndrome. However, while this test can diagnose sleep apnea syndrome, there are some limitations. Polysomnography is a labor-intensive test that requires a dedicated facility [8]. Also, sleep quality may be adversely affected by measurements takings during test [9]. In addition, polysomnography is a short-term test (1–3 days), while sleep apnea syndrome requires constant monitoring with long-term observation [10]. To tackle these problems, simpler methods should be developed that can detect sleep apnea and be used for constant monitoring. Using advanced artificial intelligence (AI), automated sleep apnea detection algorithms were developed that can easily and accurately diagnose sleep apnea syndrome from limited biological signals.

Sleep apnea causes significant changes in biological signals [11–13]. Based on these changes, there have been many studies of automated sleep apnea detection algorithms based on biological signals from limited measurements that could potentially determine the need for polysomnography or complement it. For example, sleep apnea causes changes in oxygen saturation, so there are studies that detect sleep apnea based on these changes. This led to a study that used a one-dimensional convolutional neural network (CNN) to detect sleep apnea based on a decrease in oxygen saturation [14]. Also, sleep apnea can be detected by using respiration signals [15] and derived respiration signals extracted from an electrocardiogram (ECG) [16, 17] and photoplethysmography (PPG) [18]. These studies have shown the potential to detect sleep apnea using a wearable device based on a wrist-type or Holter monitor. Deep learning methods have made huge contributions to these studies. Deep learning networks, such as CNN for images or spectrograms and long short-term memory (LSTM) for time series data can be used to analyze data from medical and healthcare sensors [19]. Accordingly, recent studies have used various signals to detect sleep apnea based on deep learning networks such as the CNN-Bidirectional LSTM and CNN-ResNet [20–22].

However, until now, sleep apnea detection algorithms have rarely considered uncertainty in classification. Without polysomnography, detecting sleep apnea based on a few biological signals can produce misclassifications due to insufficient information. From this point of view, a sample with insufficient information can be an ambiguous sample. A typical ambiguous sample is respiratory effort-related arousal (RERA). RERA is an event that does not meet the criteria for apnea or hypopnea, but that presents similar symptoms, causing arousal and decreased oxygen saturation due to upper airway resistance during sleep [23]. Biological mechanisms and symptoms of RERA can be misdiagnosed as apnea or hypopnea by traditional algorithms. Therefore, techniques for assessing the reliability and uncertainty of AI predictions for diagnosis should be considered for medical and healthcare applications [24]. When the measured confidence scores of prediction results are not high, developed AI, with the ability to reject predictions, can be very helpful in diagnosis. So, in this study, we developed an AI model capable of selective prediction by measuring uncertainty using a confidence score. There were two objectives in previous studies on selective prediction models: extracting predictive confidence scores and applying the extracted predictive confidence scores to deep learning models. Studies that extracted predictive confidence scores typically use Softmax value and Monte Carlo dropout methods [25]. Subsequently, for applying extracted confidence scores, some studies focused on how to apply confidence scores to models to increase predictive and selection capabilities simultaneously. SelectiveNet [26, 27], a state-of-art deep learning-based selective prediction model, was trained using the confidence score calculated with the selection function in the model. These studies suggested ways to reduce diagnostic errors in healthcare by rejecting predictions for low-confidence score samples and passing them on to clinicians as a second opinion or using an additional decision system for those samples only.

This study aimed to develop an algorithm that can detect sleep apnea using oxygen saturation and ECG-derived respiration (EDR) to determine the need for polysomnography or complement it. Since these signals provide insufficient information compared to polysomnography, the algorithm used selective prediction based on confidence score prediction to avoid misdiagnosis. This model captures the uncertainty of ambiguous samples and ensures classification performance with a reject option. The confidence score and rejection results were validated for ambiguous samples, such as RERA samples that are biologically similar to apnea and hypopnea. In summary, the objective of this study was to develop an automatic sleep apnea detection model that used limited biological signals to enable selective prediction based on measuring the confidence score.

## Methods & materials

### Feature extraction

The signals used in this study were EDR and oxygen saturation (SaO2), and each signal had a sampling rate of 200 Hz, which is too high to be applied to AI as raw data. Previous studies have applied the down-sampling method [28, 29]. However, if the measured signal is a high-resolution signal, the quality of the signal may be reduced by down-sampling, which may result in the removal of necessary information [30]. We used the autoencoder method as a solution. An autoencoder is a non-linear deep learning-based structure consisting of an encoder that compresses data into latent vectors and a decoder that closely reproduces the latent vectors back to the original data [31]. Our goal was to employ an encoder to extract a compressed vector and then reconstruct this vector back to the original input as closely as possible using the decoder. this process allowed us to perform dimension reduction and extract essential features while excluding unnecessary information from the SaO2 and EDR signals in all segments. By using the extracted feature, the (150,8) shaped latent vector, we successfully obtained a feature that contained information capable of accurately reconstructing the original signal.

**Fig. 1:**
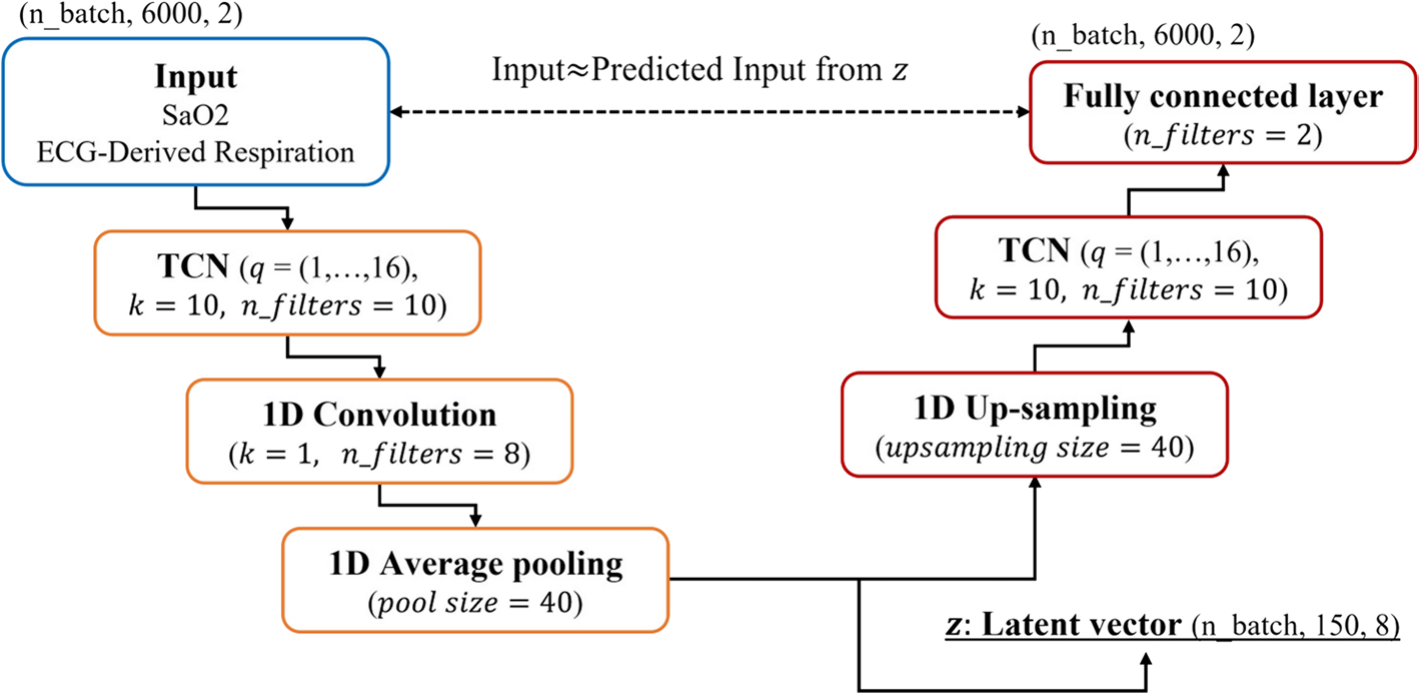
The TCN-based autoencoder structure for feature extraction

When implementing an autoencoder in this study, we designed the structure based on the temporal convolutional network (TCN) structure. A TCN is a CNN-based structure used for processing time series data by applying dilated and causal convolution [32, 33]. We used dilated convolutional layers incorporating 5 different kernel sizes, to capture patterns from local to global regions. Moreover, the utilization of causal convolutional layers enables us to retain causality by considering only past time steps, distinguishing our approach from basic CNN-based networks that compress one-dimensional signals without handling time series data. Using TCN and a 1D convolution layer, we effectively extracted features while keeping the casual characteristics of biological signals, a type of time series data. The overall structure of the autoencoder is shown in Fig. 1. An encoder consisted of the TCN and a 1D convolution layer to extract latent vector. The decoder was then structured with 1D up-sampling and a TCN structure to reproduce the original signal using a latent vector that can represent the input signal. For the TCN, we set the coefficients of dilatational convolution ($q$) to 1, 2, 4, 8, and 16 and the number of filters ($n\_filters$) to 10. For the 1D convolution, we empirically used 8 filters and set the kernel size ($k$) to 1. We calculated the loss using the mean square error (MSE) for the input and output and optimized it using Adam optimization. A trained autoencoder was used to extract the latent vectors of all the data and used as the input for classification.

### Classification & selective prediction

We used selective prediction [26] to determine the uncertainty of classification results by measuring confidence scores for the samples. Further, we provided a second option to reject prediction based on the confidence score. The prediction function $f$ performs the supervised learning for the input. The selection function $g$ is a confidence score measurement function for the input, defined as a range as follows: $g:X\to Y \left\{Y|0\le Y\le 1\right\}$ ($X$ is the input and $Y$ is the output.) When $\tau$ is the threshold for the confidence score, the selective prediction can be expressed as a combination of $f$ and $g$ as follows:

1 $$\left(f,g\right)\left(x\right)\triangleq \left\{\begin{array}{c}f\left(x\right), if\,g\left(x\right)\ge \tau .\\ don't\,know \,\left(rejection\right), otherwise.\end{array}\right.$$

This applies the prediction function $f$ for samples above the confidence score threshold, τ, and rejects prediction otherwise.

The selective prediction is controlled by variables called coverage ($\phi (g)$) and risk value ($R(f,g)$). When ${E}_{p}$ is the expected value and $\ell$ is the loss function used to converge this model, the two variables can be defined as follows:

2 $$\phi \left(g\right) \triangleq {E}_{p}\left[g\left(x\right)\right]$$

3 $$R\left(f,g\right) \triangleq \frac{{E}_{p}[\ell\left(f\left(x\right),y\right)g(x)]}{\phi (g)}$$

In the above expression, the coverage ($\phi (g)$) is the expected value of the confidence score of the sample as measured by the selection function $g$. $R(f,g)$ is the selective risk, which is the error rate for classifying the selected samples from selective prediction. Our prediction model was trained based on these two variables. We can define the empirical coverage and empirical selective risk being trained on the entire sample (${S}_{m}={\{({x}_{i},{y}_{i})\}}_{i=1}^{m}$) as follows:

4 $$\widehat{\phi }\left(g|{S}_{m}\right) \triangleq \frac{1}{m}{\sum }_{i=1}^{m}g({x}_{i})$$

5 $$\widehat{r}\left(f,g|{S}_{m}\right) \triangleq \frac{\frac{1}{m}{\sum }_{i=1}^{m}\,\ell\left(f\left({x}_{i}\right),{y}_{i}\right)g({x}_{i})}{\widehat{\phi }(g|{S}_{m})}$$

**Fig. 2:**
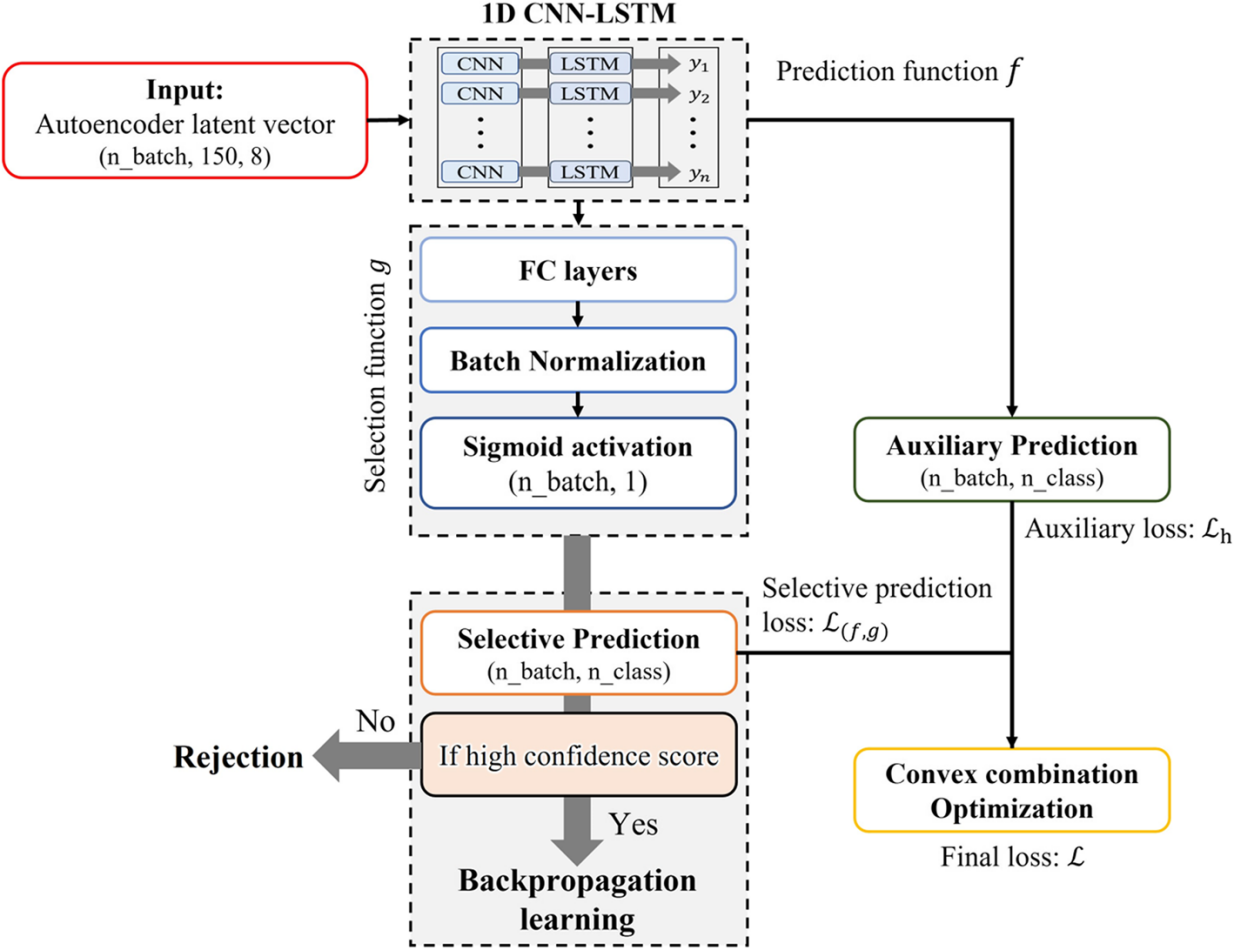
A diagram of the overall structure, including selective and auxiliary prediction

The overall structure of the implemented selective prediction is shown in Fig. 2.

This structure is divided into two parts: the selective prediction part ($(f,g)(x)$), which trains both prediction function $f$ and selection function $g$ as described earlier, and an auxiliary prediction part ($f(x)$), which assists in classification. We used a 1D CNN-LSTM [34] as a classifier $f$. The selective prediction part extracted results based on the output of the classifier, prediction function $f$, and the confidence score measured by the selection function $g$. The auxiliary prediction part contains the prediction results of the classifier. The results of the auxiliary prediction part were used to complement the results of the selective prediction part to improve the classification performance of the overall model. Both selective prediction part and auxiliary prediction part are optimized simultaneously by each of the loss functions. This will be explained in the Optimization section.

For the selection function $g$, we designed a fully connected layer, batch normalization, and a sigmoid activation layer for the output of the classifier [26]. For the prediction function $f$, our model consists of the results of a classifier and one fully connected layer.

### Optimization

Our optimization objective was to reduce the selective risk based on the confidence score for the input samples and reject prediction appropriately for samples below the confidence scores. In other words, rather than developing a model that simply memorizes the outliers of each class, we wanted to develop a model that can learn distinct attributes for each class and provide a confidence score for the classification results. For this purpose, we optimized our model by backpropagation learning only on samples that were not rejected. As a criterion for optimization, we defined a threshold for the confidence score as target coverage (*c*). The target coverage (*c*) ranges from 0 to 1. Consequently, our objective model parameters are as follows:

6 $${\theta }^{*}=arg\mathrm{min}\left(R\left({f}_{\theta },{g}_{\theta }\right)\right)\,s.t.\,\phi ({g}_{\theta })\ge c$$

We aimed to identify the model parameters that would minimize the selective risk for training samples with empirical coverage ($\phi ({g}_{\theta })$) above the target coverage ($c$). We optimized the empirical coverage ($\phi ({g}_{\theta })$) estimated by the prediction function ${f}_{\theta }$ and selection function ${g}_{\theta }$ to converge as closely as possible to the target coverage ($c$). For optimization, we used the interior point method [35] to define the loss function of the selective prediction as follows:

7 $${\mathcal{L}}_{(f,g)}\triangleq {\widehat{r}}_{\ell}\left(f,g|{S}_{m}\right)+\lambda \Psi (c-\widehat{\phi }(g|{S}_{m}))$$

8 $$\Psi \left(a\right)\triangleq \mathrm{max}{(0,a)}^{2}$$

where $c$ is the target coverage and λ is a parameter that controls the constraints of the target coverage.

The loss function has two terms. The first function (${\widehat{r}}_{\ell}$) is selective risk (Eq. 3) which is calculated for the samples selected by the section function $g$ over the input ${S}_{m}$. The second function consists of a function that is the maximum of the difference between the target coverage and the empirical coverage computed by the selection function $g$. The $\Psi$ function allows the empirical coverage to converge to the target coverage during training. We also added auxiliary loss to improve the performance of the selective prediction. The auxiliary loss was defined as the binary cross-entropy (${\mathcal{L}}_{h}$).

We trained selective prediction loss ${\mathcal{L}}_{(f,g)}$ and auxiliary prediction loss ${\mathcal{L}}_{h}$ at the same time. Both losses were optimized simultaneously based on a convex combination. Based on this, the final loss function is defined as follows:

9 $$\mathcal{L}=\alpha {\mathcal{L}}_{(f,g)}+{(1-\alpha )\mathcal{L}}_{h}$$

where $\alpha$ is a user-controlled parameter that determines the weights of the two losses.

For the specific parameter settings, the training was performed with a minibatch of 64 and a learning rate of 0.001. If the loss did not decrease, we halved the learning rate. Epochs were performed 300 times. Empirically, we set λ for the selective prediction loss to 200, and the optimal value of α for the convex combination was set to 0.3.

### Performance evaluation

In this study, we provided metrics proposed in the previous studies [36–38] and validated the selective ability of the algorithm by providing the false positive rate (type 1 errors) and the false negative rate (type 2 errors).

10 $$Accuracy=(\mathrm{TP}+\mathrm{TN})/(\mathrm{TP}+\mathrm{TN}+\mathrm{FP}+\mathrm{FN})$$

11 $$Sensitivity=\mathrm{TP}/(\mathrm{TP}+\mathrm{FN})$$

12 $$Specificity=\mathrm{TN}/(\mathrm{TN}+\mathrm{FP})$$

13 $$False\,negative\,rates=\mathrm{FN}/(\mathrm{FN}+\mathrm{TP})$$

14 $$False\,positive\,rates=\mathrm{FP}/(\mathrm{FP}+\mathrm{TN})$$

15 $$F1\,score= \frac{2TP}{2TP+ FP+FN}$$

where true positive (TP) is the number of apnea samples classified as apnea, true negative (TN) is the number of normal samples classified as normal, false positive (FP) is the number of normal samples detected as apnea, and false negative (FN) is the number of apnea samples detected as normal.

To compare the performance of selective prediction, we used the 1D CNN-LSTM model without the selection function $g$ as a baseline. We evaluated the classification performance by comparing it with the previous studies that used a large database and similar signals to our study. Furthermore, since this study was based on the multi-modality of SaO2 and EDR, we removed each signal and performed an ablation test to compare the results.

### Dataset

The dataset used in this study was polysomnography data from Massachusetts General Hospital, MGH [39]. This polysomnography data consisted of 1,983 patients with suspected sleep apnea syndrome and was composed of seven types of biological signals such as six-channel EEG, EOG, ECG, EMG (chin), SaO2, respiratory rate, and airflow with a sampling rate of 200 Hz. We used data for 994 subjects in the dataset that were annotated. The annotations for sleep apnea syndrome consisted of hypopnea (number of samples: 56,936), central apnea (22,763), mixed apnea (2,641), and obstructive apnea (32,547). In addition, this dataset was annotated at 1 s intervals for RERA (43,822), which is difficult to find in other polysomnography datasets. In this study, RERA, which is likely to be misclassified as apnea, was used as a reference for ambiguous samples, and the performance of the confidence score-based algorithm was validated. In other words, we used this dataset to see if an ambiguous sample such as RERA could avoid misdiagnosis or perform a reject option. We divided them as follows: 70% (subjects: 700) for train, 5% (50) for validation, and 25% (244) for test. Hypopnea, mixed apnea, central apnea, and obstructive apnea were grouped into one class, apnea, while other segments, excluding RERA and apnea, were grouped into another class, normal. We constructed a balanced training and test dataset, using a randomly selected dataset from normal samples for selective prediction training. This ensured that the number of samples in each class was evenly distributed during training and test.

### Pre-processing

**Fig. 3:**
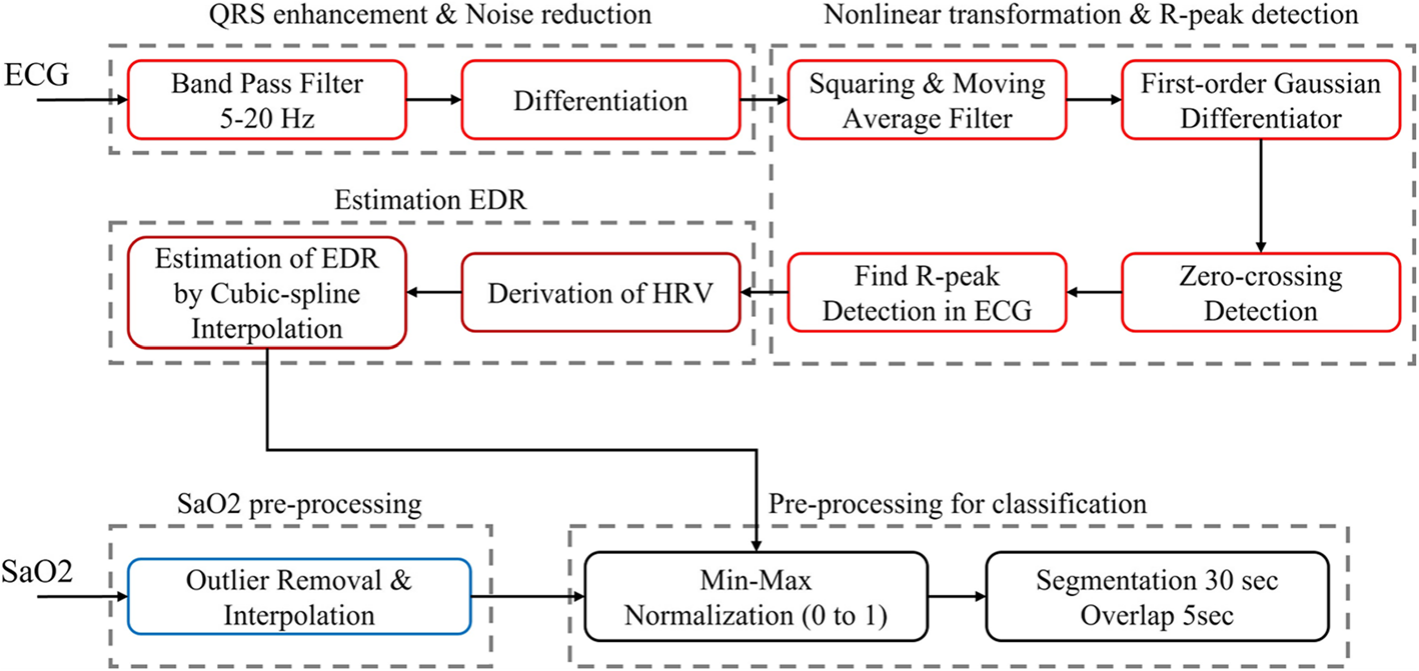
The diagrams of ECG and SaO2 pre-processing to apply to training

The preprocessing of the biological signals used in this study, ECG and SaO2, is illustrated in Fig. 3.

Robust R-peak detection was performed on the ECG to capture the QRS complex [40]. To remove the noise of ECG and enhance the QRS complex, a band pass filter was applied 5-20 Hz, and R-peak detection was performed using first order Gaussian differentiator after a nonlinear transformation. Based on the calculated RR-interval, the EDR was estimated using interpolation after calculating heart rate variability (HRV) [41]. For SaO2, outliers were removed and then compensated for by interpolation.

After pre-processing, both EDR and SaO2 were normalized to the 0–1 range for training. we performed a 30-s segmentation [21] with a 5-s overlap based on sleep apnea being longer than 10 s. After pre-processing, 701,108 samples were used for training and the remaining 220,828 samples were used for test.

## Result

### Feature extraction performance

**Fig. 4:**
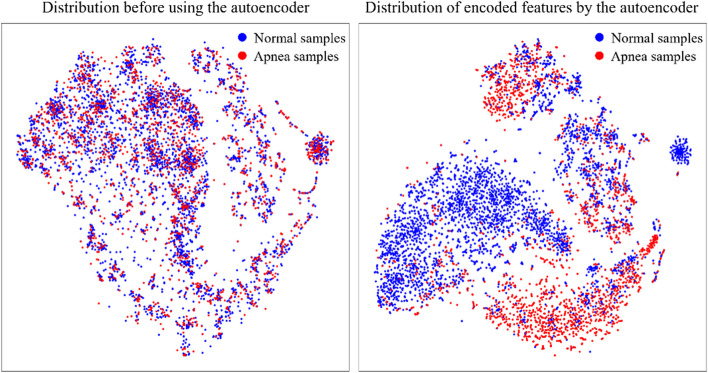
A visualization of the t-SNE results for each class input and output of autoencoder

We encoded the biological signals of SaO2 and EDR using the autoencoder method. The signals from SaO2 and EDR have a total of 12,000 samples, each containing 6,000 data points per 30 s segments. We used the autoencoder to reduce a total of 12,000 data points to 1,200. We evaluated the performance of an autoencoder that reconstructs the original signal. This algorithm was validated with a test set of 244 subjects (220,828 samples) We performed correlation analysis to determine the similarity between the reconstructed and original signals. The average correlation was 0.89. We also visualized the distribution between two classes for the latent vector extracted from the autoencoder by applying t-distributed stochastic neighbor embedding (t-SNE) [42]. Compared to the input of the autoencoder, encoded feature distributions for two classes were clustered. This visualization is shown in Fig. 4.

### Coverage violation & selective risk

We had two goals in training selective prediction. The first was to converge empirical coverage to the target coverage, and the second was to optimize the model to minimize the selective risk. Therefore, we validated the average empirical coverage, coverage violation, and selective risk on our test set to ensure that model was optimized. We defined coverage violation as the absolute mean of the difference between target coverage and empirical coverage in the entire dataset. The selective risk was the error rate of the samples selected by the model. We set the target coverage to a value that is sufficiently reliable based on previous studies [26, 27]. We validated these metrics for three different target coverage values: 0.90, 0.95, and 0.98 using 220,828 test samples. This is shown in Table 1.

**Table 1:** Empirical coverage, coverage violation, and selective risk based on target coverages

Target coverage	Average empirical coverage	Coverage violation	Selective risk
0.90	0.897	0.114	0.111
0.95	0.945	0.060	0.109
0.98	0.978	0.028	0.097

### False-positive and False-negative rate

To evaluate the performance of selective prediction, we calculated the false positive and false negative rates for the samples with high confidence scores in the test set. We also calculated the values without selective prediction. Table 2 summarizes the results for target coverage between 0.90 and 0.98 and without selective prediction.

**Table 2:** False-positive rate and False-negative rate based on target coverages

Target coverage	False-positive rate (%)	False-negative rate (%)
Without selective prediction	16.89	16.67
0.90	12.31	9.62
0.95	12.84	9.08
0.98	8.71	10.79

### Classification performance

The selective prediction was designed using a 1D CNN-LSTM for classification. We compared the classification performance with and without the selective prediction. When used with the selective prediction, the target coverage of 0.98 showed the best classification performance. Using the test set, the performance of our model was 83.22% for accuracy, 83.11% for sensitivity, 83.33% for specificity, and an F1-score of 0.832 without the selective prediction. Using the selective prediction, the accuracy was 90.26%, the sensitivity was 91.29%, the specificity was 89.21%, and the F1-score was 0.905. In summary, we could see that the selective prediction model contributed to an overall increase in performance. The performance of sleep apnea detection in previous studies and the results before and after selective prediction are shown in Table 3.

**Table 3:** Performance comparison

Study	Dataset	Method	Signal	Acc (%)	AUROC	F1-score
Sharma et al.,2022 [43]	SHHS-1 (5,793)	Feature extraction + Decision tree	SpO2	79.81	N/A	0.792
			ECG	72.31	N/A	0.710
			SpO2 + ECG + AbdoRes + ThorRes	81.63	N/A	0.812
Pragya et al.,2022 [44]	SHHS-1 (5,793)	1D CNN	SpO2 + Pulse rate	84.3	0.862	N/A
	SHH-2 (2,651)			82.2	0.904	N/A
Shanmugham et al.,2021 [21]	MGH	Feature extraction + ResNet	ECG + Respiration signal	77.00	0.840	N/A
Mahsa et al., 2021 [45]	Apnea-ECG (70)	LeNet + LSTM	ECG	80.67	N/A	0.747
Oliver et al., 2021 [46]	Apnea-ECG (70)	LSTM-CNN Hold out test	ECG (R-R interval)	81.30	85.32	N/A
Tom et al.,2018 [47]	SHHS-1 (5,793)	LSTM	ECG (EDR)	60.10	0.588	N/A
			AbdoRes	77.20	0.775	N/A
This study (Without selective prediction)	MGH (994)	1D CNN-LSTM	ECG(EDR) + SaO2	83.22	0.908	0.832
This study (With selective prediction)	MGH (994)	1D CNN-LSTM + Selective prediction	ECG(EDR) + SaO2	90.26	0.939	0.905

*Acc* Accuracy, *AUROC* Area under the curve of the receiver operating characteristic, *SHHS* Sleep heart health study database, *AbdoRes* Abdomen respiration signal, *ThorRes* Thorax respiration signal, *N/A* Not applicable, *MGH* Massachusetts general hospital database, *EDR* ECG-Derived Respiration

### Ablation test

Since we developed the multi-modality classification model using two signals (EDR and SaO2), we validated the significance of each signal for the classification. trained with either SaO2 or EDR and tested modality ablation with the target coverage of 0.98. We compared the results with and without selective prediction of each signal. The results are shown in Table 4. The classification using both SaO2 and EDR had higher classification performance than using only a single modality.

**Table 4:** Comparison of classification performance for each biological signal (with and without selective prediction)

Signal	Without selective prediction			With selective prediction		
	Acc (%)	Sen (%)	Spec (%)	Acc (%)	Sen (%)	Spec (%)
SaO2	78.49	75.83	81.15	81.17	80.32	82.12
EDR	74.76	74.33	75.21	81.36	83.96	78.58
Multi-modal	83.22	83.11	83.33	90.26	91.29	89.21

*Acc* Accuracy, *Sen* Sensitivity, *Spec* Specificity

## Discussion

### Overview

We developed a confidence score-based selective prediction using EDR and SaO2 for detecting sleep apnea. To develop selective prediction, we used a reject option to reduce the misdiagnosis rate for ambiguous samples with a low confidence score. We evaluated the performance of the developed model. First, we checked the empirical coverage and selective risk per target coverage to ensure that the trained model was optimized to be able to select samples. Based on Table 1, we have validated that the developed model has been optimized according to our desired direction. We then checked the false positive rate (type 1 error) and false negative rate (type 2 error), which are important for diagnosis in the medical field, to see the benefits of selective prediction in medical data classification. Both type 1 and type 2 error decreased after using the selective prediction. These results showed that the developed model has the potential to reduce the type 1 and the type 2 errors in sleep apnea detection. In our classification performance, we found that 0.98 is the best target coverage for classification. Based on Table 3, we found that our model showed improved performance compared to similar previous studies, and we confirmed that our model’s performance was further improved through selective prediction.

### Rejection

**Fig. 5:**
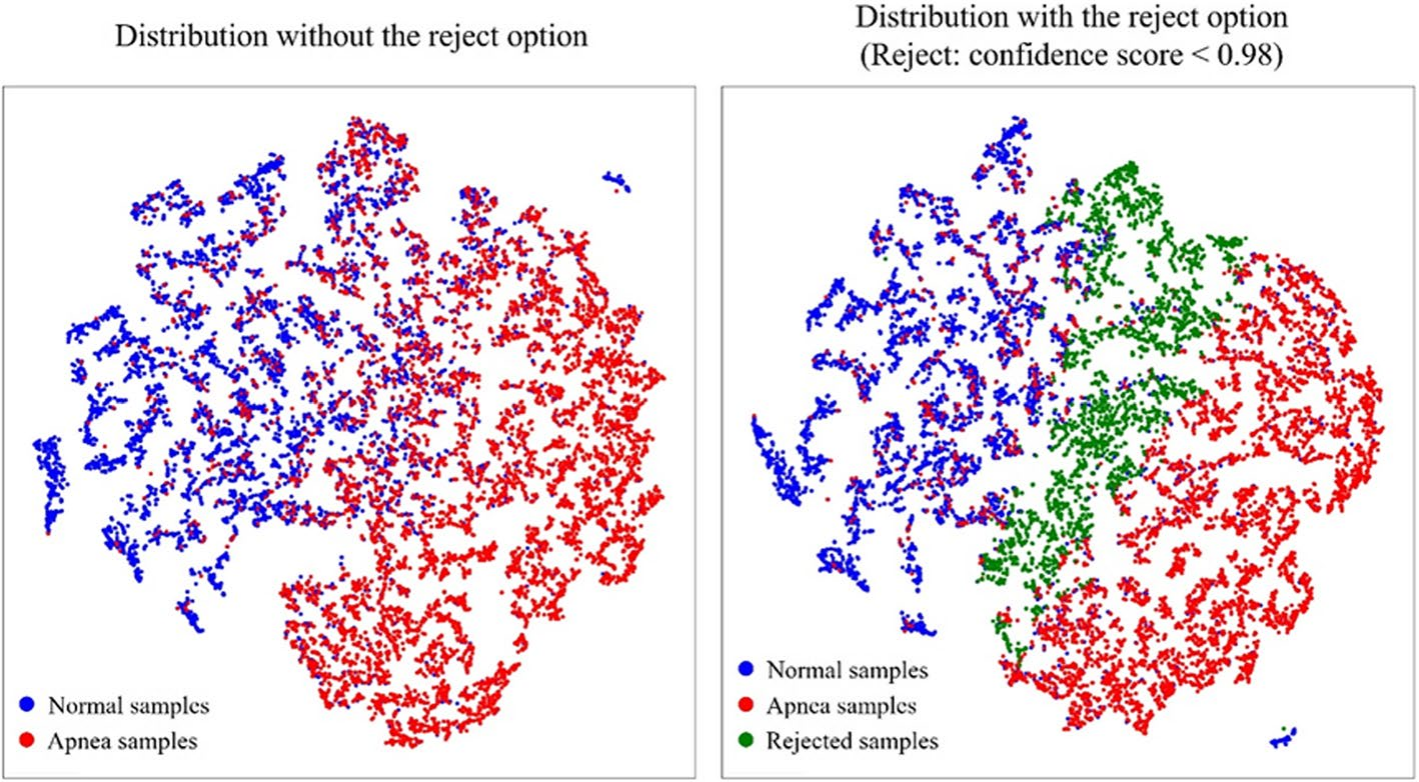
The distribution by class which were classified by selective prediction based on confidence scores

We analyzed the rejected predictions for the interpretation of the classification results. We used the output of the last dense layer of the selective prediction to visualize the apnea (subtype: obstructive apnea, central apnea, mixed apnea, hypopnea), normal, and the rejected samples. We performed a test at a 0.98 confidence score and rejected it based on the results. The result is shown in Fig. 5.

As a result, we could observe that the attributes corresponding to the apnea and normal classes form distinct clusters with each other. Also, the selective prediction rejected the samples in the area where two classes overlap because it determined those samples to be unreliable.

**Fig. 6:**
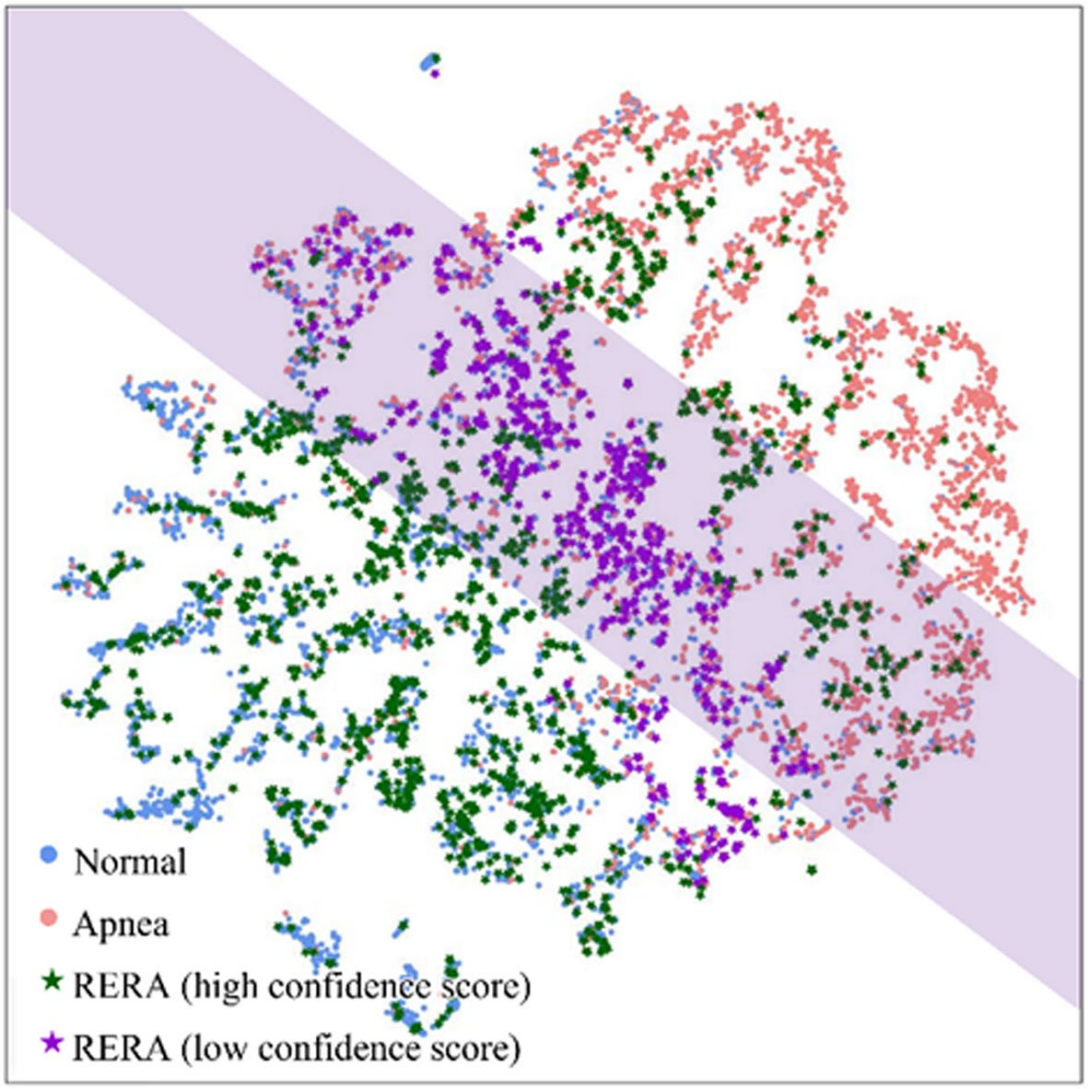
The distribution for RERA

In addition, we tested the RERA sample. As mentioned above, RERA is a symptom that is likely to be misclassified as apnea. Since we used selective prediction to reduce the error rate for ambiguous samples, we tried a test based on RERA, which biologically can be defined as a sample whose class attributes are ambiguous compared to the normal and apnea classes. As with the previous experiment, we tested at a 0.98 confidence score. As a result of the classification, 48.86% of the RERA samples were rejected, 42.81% were diagnosed as normal class samples, and only 8.33% were diagnosed as apnea class samples. In contrast, a dataset with only apnea and normal samples had 18.77% reject rate. The distribution of the RERA class compared to the distribution of apnea and normal class is shown in Fig. 6. This figure represented the distribution of apnea and normal samples, which were shown in red and blue colors, respectively. Next, we evaluated the confidence score for RERA, and if this score was less than 0.98, we classified it as a low confidence score (reject); otherwise, we classified it as a high confidence score. As shown in Fig. 6, we could observe that the classification was rejected in the purple area due to the low confidence score. These results showed that the developed model rejected a significant number of RERA class samples since these samples had less clear class attributes compared to normal and apnea samples.

Using the t-SNE visualization, our model was also able to provide interpretations for classification results by providing confidence score. In summary, based on Fig. 6 and the classification results, it could be observed that there is ambiguity in distinguishing RERA class samples from normal and apnea class samples. Due to this characteristic, using uncertainty-based classification methods such as selective prediction could be one of the ways to enhance practical applicability.

### Strengths and limitations of the study

In this study, we developed an automatic sleep apnea detection algorithm that enables selective prediction based on a confidence score using EDR and SaO2. The model used the reject option to ensure classification performance by rejecting ambiguous samples with low classification confidence. By applying the reject option, we were able to reject the classification results for samples with ambiguous class attributes. The rejected samples are then given the opportunity to be further diagnosed with a second opinion by a clinician or decision system. This can be an effective method of reducing false negatives and false positives, which can be significant in the healthcare field.

However, there are still challenges ahead to apply wearable device. We used balanced data to focus on selective prediction. So, when applying the algorithm in practice, this problem should be solved by adjusting the threshold of the receiver operating characteristic (ROC) curve [44, 48] through calculating the largest geometric mean, G-mean (G-mean $=\sqrt{sensitivity\times spectificity}$) [49].

In addition, when applying a continuous data, challenges may arise in determining the appropriate window size and handling side parts of each segment. To address these issues, we propose the utilization of sliding window and soft voting decisions, as demonstrated in a previous study [17]. By employing these techniques, we should optimize parameters such as window length and sliding window criteria to adapt the algorithm for real-world applications. In future study, it is essential to explore optimization methods to ensure practical feasibility. Therefore, our future plans involve collecting polysomnography data (DB) from sleep apnea patients using wearable devices and assessing their suitability for real-world applications. Through this study, we are optimistic that our proposed approach will significantly reduce the misdiagnosis rate when diagnosing sleep apnea, relying solely on the limited information acquired from the wearable device worn on the wrist.

## Conclusion

Selective prediction, as used in this study, proves to be a highly effective approach in mitigating false diagnoses when AI encounters significant uncertainty. To the best of our knowledge, this is the first study of automatic sleep apnea detection algorithm based on confidence scores that uses an uncertainty measure. Our study shows the potential for practical applications in wearable devices that measure biological signals, such as respiratory signals derived from ECG (EDR), photo-plethysmography and oxygen saturation. Also, we expect that the confidence score-based reject option used in this study will be a more reliable technique when applied to wearable devices that acquire low quality signal. In conclusion, our approach is expected to serve as an alert system for sleep disorders, providing a complement to polysomnography. The study will enable wearable devices to provide real-time sleep monitoring and personalized sleep quality, thus enhancing sleep management support.

## Data Availability

The datasets generated and analyzed as part of the current study are available at the physionet.org [39] repository (https://physionet.org/content/challenge-2018/1.0.0/). Our source codes used for this study are available from the GitHub repository (https://github.com/hbumjj/SelANet).

## Abbreviations

AI: Artificial intelligence
AUROC: Area under receiver operating characteristic curve
CNN: Convolutional neural network
ECG: Electrocardiogram
EDR: ECG-derived respiration
LSTM: Long short-term memory
PPG: Photoplethysmography
RERA: Respiratory effort-related arousal
TCN: Temporal convolutional network
t-SNE: T-distributed stochastic neighbor embedding
